# Barriers and enablers of implementation of alcohol guidelines with pregnant women: a cross-sectional survey among UK midwives

**DOI:** 10.1186/s12884-021-03583-1

**Published:** 2021-02-15

**Authors:** Lesley A. Smith, Judith Dyson, Julie Watson, Lisa Schölin

**Affiliations:** 1 Institute of Clinical and Applied Health Research, Faculty of Health Sciences, University of Hull, Cottingham Road, Hull, HU6 7RX UK; 2 School of Health Sciences, City, University of London, Northampton Square, London, EC1V 0HB UK; 3 Care Quality Commission, Citygate, Gallowgate, Newcastle Upon Tyne, NE1 4PA UK; 4 Nursing Studies, School of Health in Social Science, University of Edinburgh, Teviot Place, Edinburgh, EH8 9AG UK

**Keywords:** Prevention, Implementation, Behaviour change, Lifecourse epidemiology, Maternal health, Healthcare practice

## Abstract

**Background:**

In 2016, the UK Chief Medical Officers revised their guidance on alcohol and advised women to abstain from alcohol if pregnant or planning pregnancy. Midwives have a key role in advising women about alcohol during pregnancy. The aim of this study was to investigate UK midwives’ practices regarding the 2016 Chief Medical Officers Alcohol Guidelines for pregnancy, and factors influencing their implementation during antenatal appointments.

**Methods:**

Online cross-sectional survey of a convenience sample of UK midwives recruited through professional networks and social media. Data were gathered using an anonymous online questionnaire addressing knowledge of the 2016 Alcohol Guidelines for pregnancy; practice behaviours regarding alcohol assessment and advice; and questions based on the Theoretical Domains Framework (TDF) to evaluate implementation of advising abstinence at antenatal booking and subsequent antenatal appointments.

**Results:**

Of 842 questionnaire respondents, 58% were aware of the 2016 Alcohol Guidelines of whom 91% (438) cited abstinence was recommended, although 19% (93) cited recommendations from previous guidelines. Nonetheless, 97% of 842 midwives always or usually advised women to abstain from alcohol at the booking appointment, and 38% at subsequent antenatal appointments. Mean TDF domain scores (range 1–7) for advising abstinence at subsequent appointments were highest (indicative of barriers) for social influences (3.65 sd 0.84), beliefs about consequences (3.16 sd 1.13) and beliefs about capabilities (3.03 sd 073); and lowest (indicative of facilitators) for knowledge (1.35 sd 0.73) and professional role and identity (1.46 sd 0.77). Logistic regression analysis indicated that the TDF domains: beliefs about capabilities (OR = 0.71, 95% CI: 0.57, 0.88), emotion (OR = 0.78; 95%CI: 0.67, 0.90), and professional role and identity (OR = 0.69, 95%CI 0.51, 0.95) were strong predictors of midwives advising all women to abstain from alcohol at appointments other than at booking.

**Conclusions:**

Our results suggest that skill development and reinforcement of support from colleagues and the wider maternity system could support midwives’ implementation of alcohol advice at each antenatal appointment, not just at booking could lead to improved outcomes for women and infants. Implementation of alcohol care pathways in maternity settings are beneficial from a lifecourse perspective for women, children, families, and the wider community.

**Supplementary Information:**

The online version contains supplementary material available at 10.1186/s12884-021-03583-1.

## Background

Regardless of amount, alcohol consumption has an impact on health [1]. Globally, alcohol is the leading risk factor for deaths and disability-adjusted life-years (DALYs) in females aged 15–49 years, which in 2016 led to 3.8% of deaths and 2.3% of risk-attributable DALYs. Alcohol consumption over the last 10–15 years has declined in the UK, although average annual consumption is still 9.7 l of pure alcohol per adult aged at least 15 years equivalent to around 19 units per week [2]. In 2017, 14% of women exceeded 14 units per week, and 11% drank more than 6 units in 1 day in the past week, indicative of increased risk of harm [2].

The health risks associated with alcohol are liver disease, cancers, injuries and accidents and communicable diseases [1]. If alcohol is consumed during pregnancy, it may also cause miscarriage, preterm birth, low birthweight and fetal neurodevelopmental effects [3–6]. Fetal alcohol syndrome (FAS) is the more severe form of fetal alcohol spectrum disorder (FASD), which encompasses a range of cognitive, growth and neuro-behavioural impairments which have lifelong consequences [7, 8], and is estimated to cost in excess of £2 billion per year in the UK [9]. Recent meta-analyses have estimated the global prevalence of fetal alcohol effects and alcohol consumption during pregnancy. FAS was estimated as 14.6 per 10,000 livebirths [10] and FASD as 77 per 10,000 in the general population of children and youths, meaning that 1 in 13 women who consumed alcohol during pregnancy would deliver a child with FASD [11]. This is of concern, as the global prevalence of drinking in pregnancy was estimated as 9.8% (95% Confidence Interval (CI) 8.9–11.1) with the prevalence of any alcohol consumption in the UK one of the highest at 41.3% (95% CI: 32.9–49.9) [10].

In 2016, the UK Chief Medical Officers’ revised the alcohol drinking guidelines (the CMO Guidelines), and due to the lack of evidence to establish a safe level of alcohol consumption during the periconception period and during pregnancy advised complete abstinence for women who are pregnant or planning a pregnancy [12]. This brought the UK in line with many countries who already advised complete abstinence. International [13, 14] and UK [15] clinical guidelines recommend that as part of routine antenatal care all women should be assessed for alcohol consumption, provided with advice, and the appropriate level of support offered. Midwives have a key role in identifying, advising, managing and supporting women regarding alcohol consumption during pregnancy [16, 17]. Yet it is unclear to what extent midwives discuss alcohol consumption with women during antenatal appointments, whether they provide structured advice and support to change unhealthy drinking behaviours and what the barriers are to providing alcohol-related advice to pregnant women. The lack of consensus on a safe level of drinking during pregnancy may contribute to variations in practice regarding how to ask women about alcohol intake and advise them about alcohol consumption.

Studies exploring midwives’ alcohol-related knowledge and practices have been conducted in Australia [18–20], Sweden [21], Denmark [22], Norway [23], Netherlands [24], Scotland [25, 26] and in England [27]. One study showed that despite a change in policy advocating alcohol abstinence during pregnancy, only 61% of midwives implemented the advice [22]. A more recent study reported 99% of midwives advised abstinence following the same change in policy in Australia [20]. Factors that have been suggested to influence practice include tailoring of advice according to perceived risk of alcohol-related harm [19, 24]; lack of skills to discuss alcohol with women [20, 21, 23]. Time constraints, lack of organisational support [18, 20, 23], and lack of confidence in assessing and advising women about alcohol [28] have also been identified as barriers. The application of a comprehensive implementation framework to investigate determinants of practice on a large sample of midwives in the UK has not been previously reported.

There are many values to using a theoretical approach in assessing barriers to practitioner attitudes and practices. It may mitigate cognitive biases such as logic [29], automatic responses [30], and fundamental attribution error [31]. The Theoretical Domains Framework (TDF) comprises a comprehensive set of potential determinants of practice behaviour [32]. It is based on 33 published models (which include a total of 128 psychological constructs). The original 11 domains are: knowledge, skills, social/professional role and identity, beliefs about capabilities, beliefs about consequence, motivation and goals, memory attention and decision processes, environmental context and resources, social influences, emotion and action planning. A twelfth domain considers the nature of practice behaviour rather than determinants of the behaviour. More recent versions involve 14 domains with optimism, reinforcement and intentions added to the original 12 [33]. The TDF empirically maps to a number of behaviour change techniques (BCTs) that are effective in supporting or changing practice behaviours and can underpin the design of pragmatic interventions to overcome assessed barriers to implementation [34]. It has been used to address implementation of guidelines or interventions among midwives to discuss place of birth with women [35], physical activity [36] and supporting pregnant women to stop smoking [37]. Most recently, it has been used to understand midwives practices in Australia [18].

This study draws on the TDF to examine a broad range of factors that may influence midwives’ practices regarding implementation of the CMO Guidelines by UK midwives with women under their care. Specific objectives were to determine midwives knowledge of the CMO Guidelines; and to identify potential barriers and enablers of practice behaviour regarding asking and advising women who are pregnant about alcohol consumption.

## Methods

We used an anonymous self-reported online questionnaire and carried out a survey among midwives working in England, Northern Ireland, Scotland and Wales. The methods are described in greater detail in the final report [38] and are summarised below. The study is reported in line with the STROBE checklist for reporting cross-sectional studies (additional file 1).

### Sampling and recruitment strategy

The questionnaire was developed using Qualtrics, and link was distributed using social media, a project stakeholder group and the authors’ professional networks. Questionnaire data were gathered between October 2018 until January 2019. Midwives were eligible to take part if they were currently in practice in the UK. Participants had the opportunity to enter a prize draw to win one of three £100 shopping vouchers. An achieved sample size of 1000 midwives was set, similar to other questionnaire surveys of this kind, and give sufficient data for multivariable analyses based on a minimum of 300 plus at least 10 events for each variable added to the model as recommended [39–41].

### Data collection questionnaire

The questionnaire was informed by previous research on midwives alcohol-related practices [28] and the TDF [34]. Questionnaire items were discussed with a stakeholder group, comprising of academics and researchers, representatives from third sector organisations (including FASD and birthmother advocacy groups; and alcohol, maternal and infant health-related charities), midwives, public health practitioners, the Royal College of Midwives (RCM) and Public Health England (PHE). The questionnaire was pilot tested with 16 midwives and minor revisions made based on their feedback. Piloting suggested it would take 15–20 min to complete.

To determine midwives’ knowledge on alcohol-related issues, questions included knowledge of the CMO Guidelines for pregnant women. To determine practices, questions elicited information about: how and when midwives gather information on alcohol consumption, what advice is routinely given to women about alcohol consumption, whether advice is recorded in a woman’s notes and what action is taken if they are concerned about a woman’s drinking. Midwives were asked separate questions about practices regarding all women and for women whom they suspect have an alcohol problem. The knowledge questions came after the practice questions to reduce a potential order effect and all used either five-point Likert scale response categories (always, usually, occasionally, rarely, never) or free text responses.

To evaluate the determinants of midwives’ implementation of the CMO Guidelines, 26 statements aligned to 10 TDF domains were developed (see additional file 2 – Table 1). We did not include the domain ‘nature of behaviour’ here as it is not a behavioural determinant but more a set of characteristics than can be used to describe a behaviour [42]. Each of the 10 TDF domains were measured using between one and four statements. Additionally, a hypothetical statement was included “if I were pregnant now I would abstain from consuming alcohol” as a proxy for midwives’ personal attitudes towards alcohol use during pregnancy. Midwives rated their responses to all statements on a seven-point Likert scale from strongly agree to strongly disagree. Some of the statements were phrased negatively to avoid response bias [39]. TDF statements were followed by questions on alcohol education and training, demographics, and practice-related characteristics (see additional file 3 - questionnaire).

### Data analysis

Questionnaire data were transferred from Qualtrics to an Excel database, checked for fidelity, and negatively phrased questions reversed before analysis using SPSS version 25. Frequencies and percentages were calculated for categorical data. Measures of central tendency and dispersion were estimated for each TDF statement. The scores for each statement within each of the TDF domains were summed to create a domain score (range 1–7). Lower scores indicate agreement with the statement, in other words a facilitator of carrying out the behaviour, and higher scores indicate disagreement with the statement and consequently a barrier of carrying out the behaviour. As a rule of thumb, we considered scores of three or above as a barrier. Multivariable logistic regression was used to examine the relationship between each of the TDF domains and 1) at booking advising all women to abstain, and 2) other than at booking advising all women to abstain. Response categories ‘always’ and ‘usually’ were combined to represent carrying out the advice and ‘occasionally’, ‘rarely’ and ‘never’ were combined to represent a comparison group. The association was reported as an adjusted odds ratio (aOR) with 95% confidence interval (CI). All TDF domains were added as variables in the regression model.

## Results

A total of 1636 survey links were accessed, of which 957 questionnaires were completed. After exclusion of 115 surveys that did not meet the eligibility criteria, 842 were retained for analysis. The distribution of the midwives in the sample by each nation reflected the expected distribution for midwives in the UK (RCM 2016). The majority of respondents were aged > 35 years (73%), 52% had worked as a midwife for > 10 years, 43% worked in the community and a further 27% rotated between community and hospital settings. Demographic and practice characteristics of the midwives are shown in Table 1.

**Table 1:** Demographic and practice characteristics of respondents (*N* = 842)

		n	%
Location of work	England	714	85
	Northern Ireland	55	6
	Scotland	43	5
	Wales	30	4
Age	21–24	46	6
	25–34	184	22
	35–44	214	26
	45–54	251	30
	> 55	141	17
Place of work	Community or integrated team	360	43
	Hospital-based^a^	249	30
	Rotational	226	27
Where qualified	UK	790	99
	EU	6	< 1
	Outside EU	1	< 1
Years in practice	< 2 years	111	14
	3–10 years	284	34
	> 10 years	430	52

^a^Hospital-based included labour ward, day assessment unit, fetal medicine unit, post-natal ward, co-located midwife unit; rotational included midwives working in community and hospital settings and midwives with a specialist role unless community setting specified

Ninety four percent of midwives agreed with the statement that if they were currently pregnant, they would abstain from alcohol consumption.

### Knowledge regarding alcohol guidelines

Almost two thirds (58%) of midwives reported being aware of the CMO Guidelines, yet when asked what the specific recommendations are within the Guidelines, responses varied. The vast majority (91%) reported that alcohol abstinence is recommended (Table 2). However, 19% of midwives aware of CMO guidelines reported limiting intake to 1–2 units 1–2 times per week after the first trimester, avoiding intoxication (17%), and avoiding binge drinking (23%) were recommendations which align with the content of National Institute for Health and Care Excellence (NICE) antenatal guidelines pre-dating an update in 2019 [15].

**Table 2:** Awareness and perceived content of CMO guidelines

		n	%
**Aware of CMO guidelines** **(*n*** **= 832)**	Yes	484	58
	No	348	42
**Content of CMO guidelines for midwives aware of CMO guidelines (*N*** **= 484)**	Avoid alcohol completely	438	91
	Small amounts of alcohol during early pregnancy are unlikely to cause harm	173	36
	Limit to 1–2 units 1–2 times per week after first trimester	93	19
	Do not get intoxicated	81	17
	Do not binge drink	112	23
	I don’t know	1	0.2

### Alcohol assessment and advice

Midwives were asked about their usual practice regarding assessment and advice on alcohol consumption for all women (Table 3). At booking, almost all midwives ask about pre-pregnancy drinking and current frequency and quantity of alcohol consumption. In contrast, about three-quarters ask about alcohol consumption before pregnancy recognition or specifically about current heavy episodic drinking. At booking, the vast majority of midwives always (90%) or usually (7%) advise women to abstain from alcohol during pregnancy. However, only two thirds always (44%) or usually (21%) discuss the effects of drinking on mother and baby.

**Table 3:** Midwives practices regarding alcohol-related assessment and advice

			n	Always (%)	Usually (%)	Occasionally (%)	Rarely (%)	Never (%)
**All women**	**At booking**	Pre-pregnancy alcohol consumption	757	86	8	2		2
		Alcohol consumption between conception and recognition	753	62	14	8	8	8
		Current frequency	753	88	7	2	2	2
		Current quantity	753	94	4	1	0.4	0.2
		Current frequency of HED	755	56	12	13	10	9
		Advise to abstain	756	90	7	1	0.8	1
		Discuss effects of alcohol on mother and baby	741	44	21	24	9	2
	**Subsequent appointments**	Advise to abstain	812	26	12	26	24	11
		Discuss effects of alcohol on mother and baby	789	20	11	31	25	14
**Women with suspected alcohol problem**	**At booking**	Any referral for alcohol-related problem	755	75	14	5	4	2
		Family history of alcohol-related problem	753	34	17	19	16	13
		Alcohol consumption during previous pregnancies	751	53	21	10	9	8
		Context that alcohol consumption takes place	752	44	23	14	10	10
		Alcohol consumption of partner	753	44	24	16	9	7
		Advice or support to abstain or cut down	760	89	9	0.6	0.9	0.1
		Onward referral to an appropriate practitioner	762	93	6	0.4	0.5	0.1
	**Subsequent appointments**	Assess for current alcohol use	818	63	20	8	6	3
		Advise to abstain	813	67	15	8	5	4
		Discuss effects of alcohol on mother and baby	807	54	22	11	8	5
**All women**	**Any time during pregnancy (all women)**	Discuss alcohol and breast feeding	836	40	27	18	9	5
		Discuss alcohol and co-sleeping	830	77	14	5	2	2
		Discuss alcohol and parenting	831	37	21	18	14	10

At subsequent antenatal appointments, fewer midwives always (26%) or usually (12%) advise abstinence and fewer still always (20%) or usually (11%) discuss the potential alcohol-related effects on mother and baby.

Midwives were also asked about their usual practice regarding assessment and advice on alcohol consumption at booking for women with a suspected alcohol problem. The vast majority always (89%) or usually (9%) advise women to reduce or abstain from drinking and 93% always refer onward to appropriate agencies with 6% who usually refer (Table 3). However, a lower percentage of midwives always or usually further explore a woman’s drinking behaviour regarding previous referral for an alcohol-related problem and alcohol consumption during previous pregnancies, and fewer still always or usually enquire about her partners drinking and the context within that drinking takes place (see Table 3).

At antenatal appointments other than booking, the proportion of midwives who always or usually assess for current alcohol use (63 and 20%), advise to abstain (67 and 15%) and discuss alcohol effects (54 and 22%) is substantially higher if an alcohol-related problem is suspected than for all women (Table 3).

### TDF domains

Scores for each item from the TDF are shown in Table 4. For the domains beliefs about capabilities, beliefs about consequences and social influences, the mean scores were higher. This shows that these domains were considered to be stronger barriers to implementation of the CMO Guidelines compared with other domains. The individual items with higher scores (thus considered a barrier) and the TDF domains that they aligned with were: lack of belief that the guidelines are accurate and represent the best available evidence on alcohol and pregnancy (knowledge); belief that the guidelines do not support building a rapport with women (skills); that women do not like being advised about abstinence (social influences) and belief that advising women to abstain has no impact on their behaviour (beliefs about consequences). Weaker barriers were midwives’ level of confidence/self-efficacy in discussing alcohol (beliefs about capabilities), prioritising other tasks (motivation and goals) and the extent they found it rewarding (emotion). Individual items and domains with lower scores thus considered enablers of advising women to abstain were that midwives wanted to and intended to advise women about alcohol (motivation and goals) and that they see it as part of their job and agree that it is expected of them (professional role and identity).

**Table 4:** Mean domain scores (range 1–7) for questions within each domain

Domain	n	Mean (sd)
Knowledge	820	1.35 (0.73)
Social Professional Role	834	1.46 (0.77)
Motivation and goals	825	1.89 (0.92)
Skills	830	2.11 (0.63)
Memory, attention, decision process	834	2.15 (1.48)
Environment, context, resources	833	2.53 (1.29)
Emotion	822	2.72 (1.24)
Beliefs about capabilities	824	3.03 (0.73)
Beliefs about consequences	825	3.16 (1.13)
Social Influences	834	3.65 (0.84)

### Predictors of advising women to abstain from alcohol

The multivariable results for predictors of providing advice at booking are not reported here. A high proportion of midwives always or usually advised women to abstain at booking in relation to the number of potential explanatory variables added to the model. Therefore, the analysis would lack sufficient power to estimate the effect of each variable in a multivariable analysis.

The multivariable regression analysis showed that ‘beliefs about capabilities’, ‘professional role and identity’ and ‘emotion’ domains of the TDF were the determinants that were significant predictors of midwives always or usually advising abstinence at antenatal appointments other than at booking (Table 5). The likelihood of midwives advising women to abstain at subsequent appointments were significantly reduced if they did not agree that ‘providing advice was expected of them’ and ‘saw it as part of their job’ - ‘professional role and identity domain’ (aOR = 0.69, 95% CI: 0.51, 0.95). Lacking self-efficacy to inform women about alcohol consumption - ‘beliefs about capabilities domain’; not ‘feeling that it is rewarding’ and not ‘regretting to advise women’ - ‘emotions domain’ also significantly reduced the likelihood of advising women to abstain at subsequent appointments, aOR 0.71 (95% CI: 0.57, 0.88), and 0.78 (95% CI: 0.67, 0.90), respectively.

**Table 5:** Association of behavioural determinants of ‘always or usually’ advising abstinence at appointments other than booking

TDF domain	OR	95% CI	*P* value
Goals	0.90	0.68, 1.18	0.441
Beliefs about capabilities	0.71	0.57, 0.88	0.002*
Role	0.69	0.51, 0.95	0.022*
Emotion	0.78	0.67, 0.90	0.001*
Social influences	1.14	0.94, 1.39	0.188
Environmental context and resources	0.96	0.83, 1.10	0.55
Knowledge	1.06	0.84, 1.33	0.642
Skills	1.23	0.95, 1.60	0.113
Beliefs about consequences	1.14	0.97, 1.35	0.121
Memory, attention and decision	0.98	0.85, 1.12	0.724

Some midwives did not answer all questions so regression analysis involves *n* = 763; *OR* Odds ratio adjusted for all other predictors variables in the model, *CI* Confidence interval

## Discussion

This is the first study reporting on awareness and implementation of the CMO Guidelines among midwives in the UK. Around one in three midwives lacked awareness of the CMO guidelines and to some extent their content. The results indicated that the guidance recommending abstinence was not implemented at all antenatal appointments with a midwife. Practice varied according to whether the appointment was the initial or subsequent appointment, and the midwives’ perceptions of a woman’s alcohol consumption. Findings from the TDF-informed items suggested that knowledge was an enabler of practice, and that midwives see addressing alcohol consumption as part of their role and are motivated and intend to advise women. Barriers to implementing CMO guidelines were related to lack of expectation and prioritisation of gestational alcohol consumption within clinical teams, and lack of belief that it was worthwhile, rewarding or that it would lead to a beneficial change in a woman’s drinking behaviour. Midwives also lacked self-efficacy to implement the CMO guidelines. We identified professional role and identity, emotions and beliefs about capabilities as strong predictors of advising women to abstain from alcohol at appointments other than at booking, indicating these domains may be useful targets for interventions to support midwives’ implementation of alcohol assessment and advice.

The lack of awareness of both the CMO guidelines and their content was disappointing, particularly as they represented a change in 2016 from a permissive low risk stance to a precautionary abstinence stance, but perhaps not that surprising given a similar lack of awareness by the general population of the revised CMO guidelines [40]. This highlights the importance of understanding the determinants of practice behaviour as issuing guidelines in isolation are not sufficient to ensure their implementation in practice. Moreover, the NICE antenatal care guidelines [15] were in fact not updated until 3 years after the release of the CMO guidelines, potentially hindering healthcare practitioners delivering clear and consistent advice.

Despite the lack of awareness of the specific CMO guidelines, midwives advised abstinence. A limitation was that it was addressed at booking, but rarely again at subsequent antenatal appointments. This is similar to other studies examining midwives’ views and practices [18, 19, 26, 27] and women’s reported receipt of alcohol-related care [18]. This warrant further investigation since drinking behaviours may change during the course of pregnancy, and women may be more inclined to report alcohol consumption once a relationship with the midwife has developed. Furthermore, asking at each antenatal visit is a recommendation in World Health Organisation (WHO) guidelines [13]. Not addressing alcohol at subsequent appointments is a missed opportunity for the midwife to support a woman’s behaviour change.

Furthermore, we found that although advise to abstain was given, the specific risks of alcohol exposure during pregnancy were rarely discussed. This is similar to studies involving midwives in England [27] and Australia [18–20]. We found that midwives lacked confidence to inform women about the CMO guidelines, so the reason for not discussing the risks of alcohol consumption may in part be attributable to a lack of skills and knowledge to discuss wider alcohol-related health with women beyond advising them to abstain. A small qualitative study of Australian midwives reported a lack of knowledge of alcohol-related risks [19], similarly Dutch midwives reported a lack of knowledge about mechanisms and consequences of gestational alcohol consumption [24, 26]. Gilinsky (2009) found that midwives worried about giving conflicting advice about safe drinking levels. Further studies have cited time constraints, and concerns about offending women as a reason for not discussing the topic [16, 20, 25, 26, 41].

We found that beliefs about capabilities and emotions domains were important predictors of advising women to abstain at antenatal appointments other than at booking. This may be because midwives want to develop a trusted relationship with a woman and think that a woman is more likely to discuss her drinking behaviour once a relationship has been established [20, 25]. Particularly when women are not open to or are more difficult to engage in a conversation about alcohol [43]. Another potential reason for the influence of these domains is the difficulty midwives face when women report drinking before they realised they were pregnant and their desire to allay a woman’s fears about potential alcohol-related harm [44, 45]. This suggests that building skills and self-efficacy in clinical conversations about alcohol without disrupting the professional trusted relationship would be a useful component of an implementation intervention to support midwives’ practices regarding alcohol assessment and advice.

Engagement with women to promote health is in line with the public health role of the midwife in the UK and internationally [46–48]. Reassuringly, professional role and identity was a strong enabler of advising abstinence at antenatal appointments other than booking, indicating that midwives see advising women to abstain as part of their role and that it is expected of them and that they have intentions to do so. This suggests that if barriers to implementation can be overcome, then midwives would be better supported to carry out this role.

### Strengths and limitations

Strengths of this study was the application of a theoretical model to help understand factors that influence midwives advising women in line with the current national guidance on alcohol use during pregnancy, the large achieved sample size and use of an anonymous questionnaire to gather comprehensive data on midwives knowledge, practices and the behavioural determinants of practice. The convenience sampling, cross-sectional design, and reliance on self-report of compliance with the Guidelines weakens the inferences that can be drawn from the findings.

## Conclusions

The TDF has been a useful framework for identifying determinants of midwives practice behaviours regarding implementation of alcohol guidelines. The findings will help contribute to the development of a theoretically informed intervention to support midwives’ discussions with women about alcohol consumption during pregnancy. An implementation intervention to support midwives delivery of alcohol advice at each antenatal appointment and not just at booking could lead to improved outcomes for women and infants. Reducing risky drinking has benefits which extend beyond pregnancy, including during breastfeeding and subsequent pregnancies and throughout the lifecourse to prevent chronic disease. Pregnancy presents an ideal opportunity to change behaviour, and midwives are in a key position to engage with women to facilitate this change.

## Supplementary Information

**Additional file 1.**

**Additional file 2: Table S1**. Description of TDF domains in relation to the study.

**Additional file 3.**

## Data Availability

The datasets used and/or analysed during the current study are available from the corresponding author on reasonable request.

## Abbreviations

aOR: Adjusted odds ratio
BCTs: Behaviour change techniques
CMO: Chief Medical Officer
CI: Confidence Interval
DALY: Disability-adjusted life-years
FAS: Fetal alcohol syndrome
FASD: Fetal alcohol spectrum disorder
NICE: National Institute for Health and Care Excellence
PHE: Public Health England
RCM: Royal College of Midwives
STROBE: STrengthening the Reporting of OBservational studies in Epidemiology
TDF: Theoretical Domains Framework
WHO: World Health Organisation
