# Supplementary material for: Barriers and enablers of implementation of alcohol guidelines with pregnant women: a cross-sectional survey among UK midwives
Source: BMC Pregnancy Childbirth. 2021 Feb 15;21:134. doi: 10.1186/s12884-021-03583-1 (PMC7885406; doi:10.1186/s12884-021-03583-1)
Supplement: Supplementary file 2 — Additional file 2: Table S1. Description of TDF domains in relation to the study. [file 12884_2021_3583_MOESM2_ESM.docx]

| **Table 1. Description of TDF domains in relation to the study** | |  |
| --- | --- | --- |
| **Domain** | **Description** | **Survey questions** |
| **Beliefs about capabilities** | Whether advising to abstain is easy, within midwives’ control and midwives have confidence/self-efficacy to deliver the advice | - I am confident that I could advise women to abstain from alcohol during pregnancy if I wanted to - Whether or not I advise women to abstain during pregnancy is entirely up to me - I am confident that I can inform pregnant women about the CMO’s Low Risk Drinking guidelines |
| **Beliefs about consequences** | Perceived consequences of advising or failing to advise on abstinence, whether it has impact on behaviour and perceptions. Belief or lack of belief in the guidelines/evidence. | - Advising women to abstain from alcohol during pregnancy is harmful - Advising women to abstain from alcohol during pregnancy is not worthwhile - Advising pregnant women to abstain from alcohol has no impact on their behaviour - I have seen proof that pregnant women follow the advice to abstain from alcohol |
| **Environmental context and resources** | Having the resource (e.g. time, staffing level, necessary equipment) to advise women on alcohol consumption in pregnancy | - The decision to advise women to abstain from alcohol during pregnancy is beyond my control - I don't have enough time to advise pregnant women to abstain from alcohol during pregnancy |
| **Emotion** | The extent to which emotional factors (e.g. fear, anticipated regret, stress) influence midwives giving advice on alcohol consumption during pregnancy. | - Advising women to abstain from alcohol during pregnancy is not rewarding for me - I regret it if I don’t advise women to abstain from alcohol |
| **Motivation, goals and priorities** | The extent to which midwives intend to, want to and feel that the advice is worthwhile and to what extent other work tasks interfere with that | - I want to advise women to abstain from alcohol during pregnancy - I do not intend to advise women to abstain from alcohol during pregnancy      - There are other things I want to achieve in the appointment(s) with pregnant women that get in the way of asking about their alcohol use |
| **Knowledge** | Knowledge of the CMO guidelines and their content | - The CMO’s Low Risk Drinking Guidelines are accurate and represent the best evidence available on alcohol and pregnancy |
| **Memory, attention and decision processes** | Whether midwives remember to provide the advice and situations where advice is not given and | - I sometimes forget to ask women about their alcohol use |
| **Professional role and identity** | Expectations to ask about alcohol, autonomy to decide whether to ask and personal to drinking in pregnancy | - It is expected of me that I advise women to abstain from alcohol - Advising pregnant women to abstain from alcohol is part of my job - I expect to advise women to abstain from alcohol during pregnancy |
| **Skills** | If midwives have the skills/level of training required to ask and advise women about alcohol | - The CMO Low Risk Drinking Guidelines help me to build rapport with pregnant women - I have a range of communication techniques for advising pregnant women to abstain, that I can apply based on the needs of the woman - It is hard for me to advise women to abstain from alcohol during pregnancy |
| **Social influences** | Perceptions about whether women like/dislike the midwife’s advice and pressure/support/expectations from colleagues and superiors | - My colleagues think I should advise women to abstain from alcohol during pregnancy - I feel under pressure from my colleagues to advise women to abstain from any alcohol during pregnancy - Women don't like it when I tell them to abstain from alcohol - My senior colleagues consider it important that I advise pregnant women to abstain from alcohol |
