# Supplementary material for: Barriers and enablers of implementation of alcohol guidelines with pregnant women: a cross-sectional survey among UK midwives
Source: BMC Pregnancy Childbirth. 2021 Feb 15;21:134. doi: 10.1186/s12884-021-03583-1 (PMC7885406; doi:10.1186/s12884-021-03583-1)
Supplement: Supplementary file 3 — Additional file 3. [file 12884_2021_3583_MOESM3_ESM.pdf]

Questionnaire

Thank you for taking the time to follow this link. Answering questions 01 and 02 is required in order to determine your eligibility to participate in the survey.

The questionnaire should take you about 15–20 minutes to complete.

Although some questions might seem similar or repetitive, your responses are important to us because we want to understand the experiences of a large, representative sample of midwives when discussing alcohol with pregnant women at different times.

As a token of our appreciation for completing the questionnaire your name will be entered into a prize draw for a chance to win 1 of 3 £100 John Lewis vouchers. Please enter your contact details at the end of the questionnaire if you want to be included in the prize draw.

The information you give will not be made available to anyone who is not a member of the research team. All information you give will be anonymous and treated as strictly confidential. Please try to answer all the questions as accurately as possible

Please tick this box if you are happy taking part in the survey ❑

---

01. Are you currently working as a midwife?

❑ Yes
❑ No

02. Where are you working as a midwife?

❑ England
❑ Wales
❑ Scotland
❑ Northern Ireland
❑ Outside the UK

If response to question 01 and/or 02 is no/outside the UK, the following text will be displayed:

Thank you for your interest in taking part in this survey, however at this time we are only looking for midwives who are currently practicing as midwives inside the UK.

Section 1: Practices

This section asks you to tell us what information you gather from women and what you advise them about alcohol during pregnancy. Please note that some questions refer to all women whilst others refer only to a particular group of women, as specified.

1. At booking, please indicate the extent to which you gather information on the following factors from all women regarding alcohol consumption during pregnancy (Please tick one option from each row)

	Always	Usually	Occasionally	Rarely	Never
Drinking behaviour before the current pregnancy	❑	❑	❑	❑	❑
Drinking behaviour during the period between conception (if known) and recognition of the current pregnancy	❑	❑	❑	❑	❑
Quantity of current alcohol intake	❑	❑	❑	❑	❑
Frequency of current alcohol intake	❑	❑	❑	❑	❑
Frequency of current 'binge' drinking (drinking > 6 units on a single drinking occasion)	❑	❑	❑	❑	❑

2. At booking, please indicate the extent to which you provide the following advice to all women regarding alcohol consumption in pregnancy? (Please tick one option from each row)

	Always	Usually	Occasionally	Rarely	Never
To abstain from drinking alcohol	❑	❑	❑	❑	❑
To avoid drinking alcohol in the first 3 months of pregnancy	❑	❑	❑	❑	❑
It is OK to drink 1–2 units once or twice per week	❑	❑	❑	❑	❑
To avoid binge drinking (>6 units on a single drinking occasion)	❑	❑	❑	❑	❑
No specific advice	❑	❑	❑	❑	❑
Discuss the effects of alcohol on mother and baby	❑	❑	❑	❑	❑

3. Other than at booking, how often do you advise or do the following, with all women? (Please tick one option from each row)

	Always	Usually	Occasionally	Rarely	Never
To abstain from drinking alcohol	❑	❑	❑	❑	❑
To avoid drinking alcohol in the first 3 months of pregnancy	❑	❑	❑	❑	❑

It is OK to drink 1–2 units once or twice per week	❑	❑	❑	❑	❑
To avoid binge drinking (>6 units on a single drinking occasion)	❑	❑	❑	❑	❑
No specific advice	❑	❑	❑	❑	❑
Discuss the effects of alcohol on mother and baby	❑	❑	❑	❑	❑

4. At booking, please indicate the extent to which you gather information on the following factors <u>when you suspect that alcohol may be a problem</u> (Please tick one option from each row)

	Always	Usually	Occasionally	Rarely	Never
Any referral for a history of alcohol use	❑	❑	❑	❑	❑
Family history of alcohol abuse or dependence	❑	❑	❑	❑	❑
Drinking behaviour during previous pregnancies, if relevant	❑	❑	❑	❑	❑
Context that drinking takes place (e.g. alone/socially)	❑	❑	❑	❑	❑
Drinking behaviour of partner	❑	❑	❑	❑	❑

5. Other than at booking, how often do you do or advise the following, <u>when you suspect that alcohol may be a problem</u>? (Please tick one option from each row)

	Always	Usually	Occasionally	Rarely	Never
Assess for current alcohol use	❑	❑	❑	❑	❑
To abstain from drinking alcohol	❑	❑	❑	❑	❑
It is OK to drink 1–2 units once or twice per week	❑	❑	❑	❑	❑
To avoid binge drinking (>6 units on a single drinking occasion)	❑	❑	❑	❑	❑
No specific advice is given	❑	❑	❑	❑	❑
Discuss the adverse effects of alcohol on mother and/or baby	❑	❑	❑	❑	❑

6. At booking, if alcohol is identified as a problem, before or during pregnancy, how likely is it that you would do the following? (Please tick one option from each row)

	Always	Usually	Occasionally	Rarely	Never
Provide advice or support about cutting down or abstaining	❑	❑	❑	❑	❑

Onward referral to an appropriate practitioner/agency e.g. GP, Drinkline	❑	❑	❑	❑	❑
Complete a vulnerability pathway	❑	❑	❑	❑	❑
Take no action	❑	❑	❑	❑	❑

7. At any time during pregnancy, please indicate the extent to which you discuss the following with <u>all women</u> about alcohol use post-birth (Please tick one option from each row)

	Always	Usually	Occasionally	Rarely	Never
Recommendations about drinking alcohol when breastfeeding	❑	❑	❑	❑	❑
Alcohol and parenting	❑	❑	❑	❑	❑
Alcohol and co-sleeping	❑	❑	❑	❑	❑

8. If you enquire about alcohol consumption with pregnant women, please indicate if you use any of the following screening tools (Please tick all that apply)

❑ AUDIT
❑ AUDIT-C
❑ T-ACE
❑ TWEAK
❑ CAGE
❑ MAST
❑ Any other (specify) ____________
❑ I do not use a named screening tool

9. Are you aware of the recommendations about alcohol and pregnancy stated in the Chief Medical Officer's (CMO's) Low Risk Drinking Guidelines?

❑ Yes
❑ No

10. To your knowledge, what is/are the recommendation(s) regarding alcohol consumption for pregnant women in the CMO's Low Risk Drinking Guidelines? (Please tick all that apply)

❑ Avoid alcohol all together
❑ If you have drunk small amounts in early pregnancy, it is unlikely that it has caused harm
❑ If you drink, limit yourself to 1–2 units once or twice per week after the first trimester
❑ Do not get intoxicated
❑ Do not binge drink (>6 units in one occasion)
❑ I don't know

11. Please indicate to what extent you agree with the following statement:

The CMO's Low Risk Drinking Guidelines are accurate and represent the best evidence available on alcohol and pregnancy

Strongly agree						Strongly disagree
❑	❑	❑	❑	❑	❑	❑

Section 2: Views on asking pregnant women about alcohol and providing advice

In this section we are interested in finding out about your views on advising women on drinking alcohol whilst pregnant. Some of the questions may seem repetitive; however they each provide unique and important information.

12. For each statement below, please indicate your response on the 7-point rating scale going from 'strongly agree' to 'strongly disagree'.

12a. It is expected of me that I advise women to abstain from alcohol

Strongly agree						Strongly disagree
❑	❑	❑	❑	❑	❑	❑

12b. Advising women to abstain from alcohol during pregnancy is harmful

Strongly agree						Strongly disagree
❑	❑	❑	❑	❑	❑	❑

12c. I want to advise women to abstain from alcohol during pregnancy

Strongly agree						Strongly disagree
❑	❑	❑	❑	❑	❑	❑

12d. I am confident that I could advise women to abstain from alcohol during pregnancy if I wanted to

Strongly agree						Strongly disagree
❑	❑	❑	❑	❑	❑	❑

12e. Advising women to abstain from alcohol during pregnancy is not rewarding for me

Strongly agree						Strongly disagree
❑	❑	❑	❑	❑	❑	❑

12f. My colleagues think I should advise women to abstain from alcohol during pregnancy

Strongly agree						Strongly disagree
❑	❑	❑	❑	❑	❑	❑

12g. I do not intend to advise women to abstain from alcohol during pregnancy

Strongly agree						Strongly disagree
❑	❑	❑	❑	❑	❑	❑

12h. I feel under pressure from my colleagues to advise women to abstain from any alcohol during pregnancy

Strongly agree						Strongly disagree
❑	❑	❑	❑	❑	❑	❑

12i. The decision to advise women to abstain from alcohol during pregnancy is beyond my control

Strongly agree						Strongly disagree
❑	❑	❑	❑	❑	❑	❑

12j. I expect to advise women to abstain from alcohol during pregnancy

Strongly agree						Strongly disagree
❑	❑	❑	❑	❑	❑	❑

12k. Advising women to abstain from alcohol during pregnancy is not worthwhile

Strongly agree						Strongly disagree
❑	❑	❑	❑	❑	❑	❑

12l. It is hard for me to advise women to abstain from alcohol during pregnancy

Strongly agree						Strongly disagree
❑	❑	❑	❑	❑	❑	❑

12m. Whether or not I advise women to abstain during pregnancy is entirely up to me

Strongly agree						Strongly disagree
❑	❑	❑	❑	❑	❑	❑

12n. Advising pregnant women to abstain from alcohol will not prevent alcohol-related birth defects

Strongly agree						Strongly disagree
❑	❑	❑	❑	❑	❑	❑

12o. Advising pregnant women to abstain from alcohol has no impact on their behaviour

Strongly agree						Strongly disagree
❑	❑	❑	❑	❑	❑	❑

12p. I have seen proof that pregnant women follow the advice to abstain from alcohol

Strongly agree						Strongly disagree
❑	❑	❑	❑	❑	❑	❑

12q. Advising pregnant women to abstain from alcohol is part of my job

Strongly agree						Strongly disagree
❑	❑	❑	❑	❑	❑	❑

12r. I sometimes forget to ask women about their alcohol use

Strongly agree						Strongly disagree
❑	❑	❑	❑	❑	❑	❑

12s. There are other things I want to achieve in the appointment(s) with pregnant women that get in the way of asking about their alcohol use

Strongly agree						Strongly disagree
❑	❑	❑	❑	❑	❑	❑

12t. I regret it if I don’t advise women to abstain from alcohol

Strongly agree						Strongly disagree
❑	❑	❑	❑	❑	❑	❑

12u. Women don’t like it when I tell them to abstain from alcohol

Strongly agree						Strongly disagree
❑	❑	❑	❑	❑	❑	❑

12v. I have a range of communication techniques for advising pregnant women to abstain, that I can apply based on the needs of the woman

Strongly agree						Strongly disagree
❑	❑	❑	❑	❑	❑	❑

12x. My superiors consider it important that I advise pregnant women to abstain from alcohol

Strongly agree						Strongly disagree
❑	❑	❑	❑	❑	❑	❑

13c. I am confident that I can inform pregnant women about the CMO's Low Risk Drinking guidelines

Strongly agree						Strongly disagree
❑	❑	❑	❑	❑	❑	❑

14b. I never have enough time to advise pregnant women to abstain from alcohol during pregnancy

Strongly agree						Strongly disagree
❑	❑	❑	❑	❑	❑	❑

15. Please indicate the degree to which you agree with the below statement about the CMO drinking guidelines:

15a. The CMO Low Risk Drinking Guidelines help me to build rapport with pregnant women

Strongly agree						Strongly disagree
❑	❑	❑	❑	❑	❑	❑

16. Are there any barriers that prevent or inhibit you from advising pregnant women to abstain from alcohol?

❑ Yes
❑ No

16a. If yes, please outline below what those barriers are: ____________

17. Are there any situations or reasons where you do not advise pregnant women to abstain from alcohol?

❑ Yes
❑ No

17a. If yes, what are the situations or reasons?: ___________

18. Please indicate to what degree you agree with the following, hypothetical, statement: If I got pregnant now, I would not drink any alcohol during my pregnancy

Strongly agree						Strongly disagree
❑	❑	❑	❑	❑	❑	❑

Section 3: Education, training and resources

19. Is there a nominated person for alcohol in antenatal care in your health board/trust?

❑ Yes
❑ No
❑ I don't know

20. Do Midwifery Support Workers work alongside you in your health board/trust?

❑ Yes
❑ No

21. As part of which midwifery programme was your education on alcohol use during pregnancy provided? (Tick all that apply)

❑ Undergraduate
❑ Pre-qualification
❑ Postgraduate
❑ Post-qualification (if not ticked skip questions about post-qualification training)
❑ None of the above (skip to later question)

22. Who provided the education sessions on alcohol use during pregnancy that you received before you started working as a midwife? (Tick all that apply)

❑ Higher Education Provider i.e. a university
❑ School of Nursing or Midwifery
❑ National Health Service (NHS)/Health and Social Care (HSC) or Primary Care Trust (PCT)/Health Board
❑ Local Authority e.g. Drug and Alcohol Team (DAAT)
❑ Local or national charity e.g. Alcohol Concern, National Organisation on Fetal Alcohol Syndrome
❑ A private company e.g. Diageo

23. In total, how many hours of education on alcohol use during pregnancy did you receive before you started working as a midwife?

❑ None
❑ <2 hours
❑ 2–4 hours
❑ 5–8 hours
❑ 9-16 hours
❑ >16 hours

24. Who provided the education sessions on alcohol use during pregnancy that you received after you started working as a midwife? (Tick all that apply)

❑ Higher Education Provider i.e. a university
❑ School of Nursing or Midwifery
❑ National Health Service (NHS)/Health and Social Care (HSC) or Primary Care Trust (PCT)/Health Board
❑ Local Authority e.g. Drug and Alcohol Team (DAAT)
❑ Local or national charity e.g. Alcohol Concern, National Organisation on Fetal Alcohol Syndrome
❑ A private company e.g. Diageo
❑ I don't know/I can't remember
❑ Other (specify): ____________

25. In total, how many hours of education on alcohol use during pregnancy did you receive after you started working as a midwife?

❑ None
❑ <2 hours
❑ 2–4 hours
❑ 5–8 hours
❑ 9-16 hours
❑ >16 hours
❑ I don't know/can't remember

26. Are you offered regular (e.g. annual) training updates on alcohol and pregnancy?

❑ Yes
❑ No

27. Please list resources that you can access to give to pregnant women on alcohol consumption during pregnancy and/or foetal alcohol spectrum disorder:

____

28. Is there a system in place where you record if you have advised pregnant women not to drink any alcohol? (Tick all that apply)

❑ Yes, paper-based records
❑ Yes, electronic records
❑ No

28a. How often do you record the advice you have given to pregnant women (please tick one)

Always	Usually	Occasionally	Rarely	Never
❑	❑	❑	❑	❑

Section 4: Demographics

29. What is your age group?

❑ 21–24
❑ 25–34
❑ 35–44
❑ 45–54
❑ 55–64
❑ >65

30. What is your ethnic group?

❑ White (English)
❑ White (Welsh)
❑ White (Scottish)
❑ White (Norther Irish)
❑ White (Other)
❑ Irish Traveller
❑ Mixed/ Multiple ethnic groups
❑ Asian/ Asian British
❑ Black/ African/ Caribbean/ Black British
❑ Chinese
❑ Arab
❑ Other ethnic group

31. Where did you gain your midwifery qualification?

❑ UK
❑ European Union (EU)
❑ Elsewhere

32. In what year did you qualify as a midwife? (YYYY): ________

33. For how many years since you qualified have you worked as a midwife?: ___________

34. In which clinical setting do you currently work? (Tick all that apply)

❑ Hospital labour ward
❑ Hospital antenatal or postnatal ward
❑ Hospital antenatal clinic
❑ Midwifery-led unit attached to or inside a hospital
❑ Standalone midwifery-led unit in the community
❑ Community (not including a midwifery led unit)
❑ Rotational post
❑ Other: _________________

36. When did you last carry out a booking?

- ❑ Within the last week
- ❑ Within the last month
- ❑ Within the last year
- ❑ More than 1 year ago

37. What is the first two letters of the postcode of where you practice? (this is just for enable us to contrast the results between different areas): _____________
